# Characterization of the Oral Microbiome and Anticipated Functional Profiles of Companion Animals in Private and Cohabiting Environments: A Pilot Study

**DOI:** 10.3390/ani16121882

**Published:** 2026-06-17

**Authors:** Charinya So-In, Nisachon Chaowang, Phimchaya Srisomporn, Phiramada Anu-an, Supreeya Paiboon, Sirinan Thananchai, Charinthip Ninolo, Phitcharat Sunthamala, Sujira Maneerat, Sunanta Chuncher, Priyapa Najomtien, Surasak Khankhum, Nuchsupha Sunthamala

**Affiliations:** 1 Department of Veterinary Technology, Faculty of Agricultural Technology, Kalasin University, Kalasin 46000, Thailand; charinya.pi@ksu.ac.th; 2 Mahasarakham University Demonstration School (Secondary), Mahasarakham 44150, Thailand; 3 Department of Biology, Faculty of Science, Mahasarakham University, Mahasarakham 44150, Thailand; 4 Department of Biotechnology, Faculty of Technology, Mahasarakham University, Mahasarakham 44150, Thailand; 5 Epidemic Simulation and Aetiology Nexus for Infectious Diseases, Mahasarakham University, Mahasarakham 44150, Thailand

**Keywords:** companion animals, microbiome convergence, host–microbe interaction, 16S rRNA sequencing, microbial community structure

## Abstract

The intricate interaction of a host’s microbiome, the microbiomes of other hosts, and environmental microbial populations significantly impacts host health, given the essential physiological functions the microbiome performs within the organism. The oral microbiome of domesticated animals is also influenced by a variety of host and environmental factors. This study investigated the characteristics of the oral microbiome of dogs and cats under comparable and disparate living conditions, emphasizing the description of diversity patterns, taxonomic composition, and predicted functional profiles. Oral buccal swabs were collected from four groups of companion animals (*n* = 5 per group): dogs housed alone in single-pet households (Group A), dogs cohabiting with cats in multi-pet households (Group B), cats cohabiting with dogs from the same households (Group C), and cats housed alone in single-pet households (Group D). The cohabiting groups were derived from five multi-pet households, with one dog and one cat sampled from each household. Amplicon sequence variations (ASVs) were used for downstream analysis after 16S rRNA gene sequencing. Rarefaction curve behavior indicated proper sequencing depth. Alpha diversity varied by group (Shannon index, *p* = 0.045), with Groups C and D having larger diversity. A Beta diversity study revealed community composition differences (Bray–Curtis dissimilarity, R^2^ = 0.257, *p* = 0.001), with some overlap between groupings. In all samples, *Proteobacteria*, *Firmicutes*, *Bacteroidota*, and *Fusobacteriota* dominated the microbiome. The relative abundance of Fusobacterium, Porphyromonas, and Pasteurella varied across groups. Core microbiome analysis identified limited overlap of core ASVs between groups, with most taxa being group-specific. Functional prediction using PICRUSt2 suggested differences in predicted metabolic and cellular pathways. Overall, these exploratory findings suggest that the oral microbiome of companion animals may be influenced by host species and cohabitation conditions. Although limited by the small sample size, the study provides preliminary insights into microbial diversity, community structure, and predicted functional profiles that may inform future One Health-oriented investigations.

## Simple Summary

Oral microorganisms play a crucial role in animal health, but the various factors influencing these microorganisms in companion animals still require further study to achieve a complete understanding. This study investigated the oral microbiome of dogs and cats under various housing environments, including solitary and communal living arrangements. The 16S rRNA gene sequencing was utilized to analyze microbial diversity, community composition, and anticipated functional characteristics among different groups. Variations in microbial diversity and composition were identified with some overlap between groups. Various bacterial species exhibited fluctuations in relative abundance based on housing conditions. Analysis of the core microbiome identified prevalent and group-specific microbial organisms. Moreover, functional predictions revealed diversity in metabolic and cellular processes. Although these findings are based on computational inference, they indicate possible variations in nutrition processing and environmental adaptability among the groups. Overall, this study provides a comprehensive baseline of oral microbiome patterns in dogs and cats, serving as a foundation for future research on companion animal health and the microbial dynamics of shared environments.

## 1. Introduction

Microbial communities in the oral cavity are numerous, varied, and complicated, and they are essential to both the health and illness of humans and animals [1,2]. The oral environment is composed of both hard and mucosal surfaces (e.g., the tongue, lips, buccal mucosa, palate, and teeth) that are perpetually moistened by saliva and, in certain regions, gingival fluid, which supplies nutrients to the oral microorganisms. The tooth enamel facilitates the growth and development of intricate biofilms, which undergo changes in their properties over the course of an individual’s life. The oral cavity is not only the initial part of the digestive tract, but also a complex and biodiverse ecosystem that is home to a tremendous number of microorganisms. *Firmicutes*, *Proteobacteria*, *Bacteroidota*, and *Actinobacteria* are the primary bacterial groups that are frequently observed in the mouths of animals, according to research. Nevertheless, the microorganisms are specialized to the animal species [3]. *Porphyromonas* and *Moraxella* are the most frequently encountered bacteria in canines, for instance. *Neisseria* and *Pasteurella* are more prevalent in cats, whereas rabbits have a distinct microbial structure that primarily includes *Streptococcus* and *Staphylococcus*. These distinctions indicate that the oral environment of each animal species is distinct from that of the human oral microbiome, which is conducive to microbial proliferation [1,4,5].

Oral diseases, particularly periodontal disease, are the result of dysbiosis, a condition in which the equilibrium of these microorganisms is disrupted. In this condition, the number of aerobic bacteria decreases and is replaced by anaerobic, Gram-negative bacteria with pathogenic properties, such as *Porphyromonas* species [6]. This alteration in the oral microbial community, characterized by an increased abundance of anaerobic pathogenic bacteria, often leads to chronic inflammation, gum tissue damage, and may negatively affect the overall health of the pet. The zoonotic potential is a particularly concerning, as it involves the possibility that healthy animals may be latent carriers of specific pathogens, such as *Staphylococcus pseudintermedius*, which is frequently reported to exhibit multidrug resistance, particularly to penicillin [7,8]. These bacteria are not merely residents of the oral cavity, but they are essential in assessing the health of companion animals. The predominant category, *Firmicutes*, encompassing *Streptococcus* strains, is a fundamental group found in almost all animal species. These bacteria act as pioneers, adhering to tooth surfaces and creating a suitable environment for other microorganisms. Meanwhile, proteobacteria, particularly in dogs, are extremely important in terms of biodiversity, as they often make up over 20% of saliva. Bacteria like *Neisseria* play a role in oral metabolism, and an imbalance in their balance can lead to changes in the pH of saliva. Under normal conditions, members of the phyla *Bacteroidota* and *Actinobacteriota* are commonly present within the oral microbiota. As dental plaque accumulates and biofilm complexity increases, localized oxygen depletion may occur, promoting the proliferation of anaerobic taxa such as members of the phylum *Bacteroidota* [9,10].

Given the complexity and heterogeneity of household environments in companion animals, this study was designed as a controlled exploratory pilot investigation focusing on privately owned animals with well-characterized housing conditions and close-contact environments. This reflects the fact that maintaining oral hygiene in pets and exercising good hygiene practices in close contact with them is not just about animal health, but also crucial in preventing the spread of antibiotic-resistant infections to humans. Therefore, understanding the oral microbiome of animals helps to highlight the connection between biodiversity, microbial balance, and the long-term health of both animals and humans.

## 2. Materials and Methods

### 2.1. Sample Collection and Experimental Design

This study included 20 companion animals distributed across four groups (*n* = 5 per group) according to species and housing condition. Group A consisted of dogs living in a household where only dogs were present, whereas Group D consisted of cats living in a household where only cats were present. Groups B and C were recruited from five multi-pet households in which dogs and cats cohabited within the same indoor environment. One dog from each household was assigned to Group B and one cat from the same household was assigned to Group C, resulting in paired dog–cat samples from shared living environments. Because only five cohabiting households were available for enrollment, the study was designed primarily as an exploratory ecological comparison rather than a household-level inferential analysis.

To reduce environmental and host-related variability, only clinically healthy indoor companion animals from privately owned households with stable and clearly documented cohabitation patterns were included. All animals were maintained under indoor housing conditions, received species-appropriate commercial dry diets, and had no recent history of antibiotic administration. Animals with clinically apparent periodontal disease, gingivitis, systemic illness, or recent antibiotic exposure were excluded. Information regarding previous professional dental cleaning was not systematically available and therefore could not be used as an exclusion criterion. Detailed demographic and husbandry information, including species, sex, age, housing condition, and dietary management, is provided in Table 1.

Oral microbiome samples were collected from the buccal mucosa using sterile swabs. To minimize variation associated with recent food intake, all samples were collected in the morning before feeding using a standardized sampling protocol and processed under identical laboratory conditions. Due to the strict inclusion criteria and the cost-intensive nature of microbiome sequencing analyses, the study cohort was intentionally limited and designed as an exploratory pilot dataset.

Samples were collected using sterile cotton swabs from the buccal mucosa and immediately transferred into sterile collection tubes for DNA extraction under standardized conditions to minimize environmental and handling variability. To mitigate technical bias, all samples were handled concurrently utilizing similar methods. Negative controls were incorporated during PCR amplification to detect possible contamination. This research was conducted under the supervision of a licensed veterinarian and adhered to a proposal approved by the Institutional Animal Care and Use Committee at Mahasarakham University, Thailand (No. IACUC-MSU-55/2023). Because the study involved only non-invasive oral swab collection, DNA extraction, and culture-independent 16S rRNA gene sequencing without microbial cultivation or genetic manipulation, institutional biosafety committee approval was not required under institutional guidelines.

### 2.2. DNA Extraction and Bacterial DNA Quality Assessment

**Figure 1:**
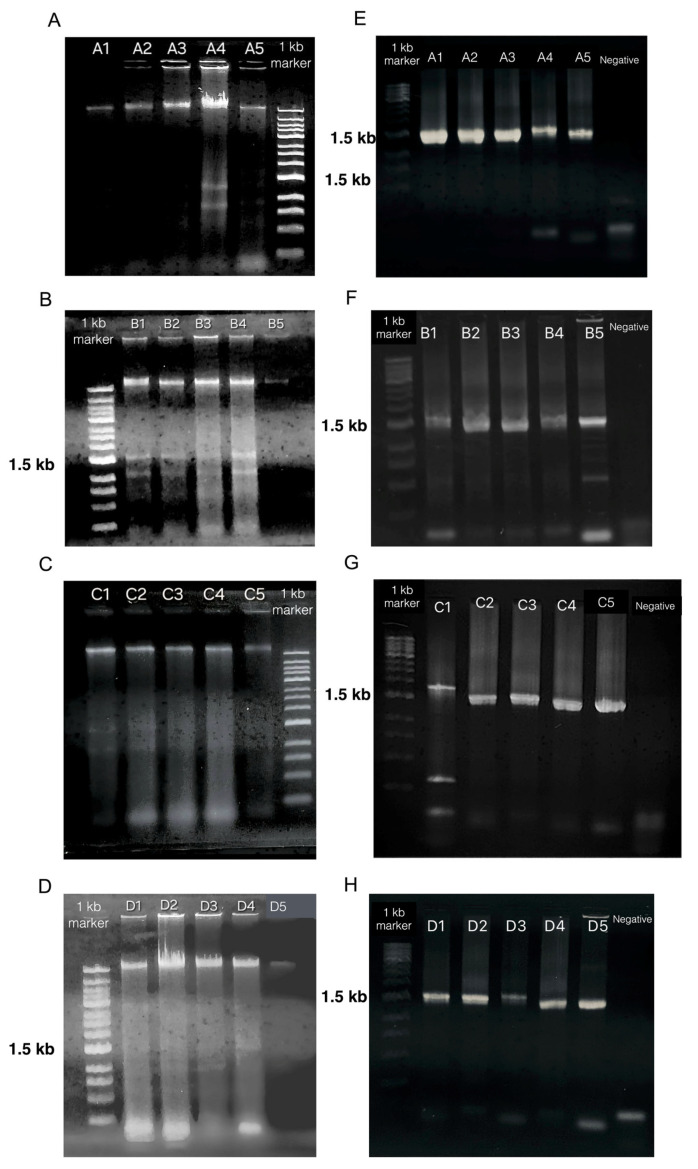
Bacterial DNA and PCR amplification of 16S rRNA gene across all samples. (**A**–**D**) Agarose gel electrophoresis of bacterial DNA from samples in Groups A–D, respectively. Lanes A1–A5, B1–B5, C1–C5, and D1–D5 represent individual samples within each group. A 1 kb DNA ladder was used as a molecular size marker. (**E**–**H**) Representative gels showing 16S rRNA gene amplification consistency across samples, including negative controls. A clear band at approximately ~1.5 kb was observed in all samples, confirming successful amplification of the full-length 16S rRNA gene. Negative controls showed no amplification, indicating absence of contamination.

Microbial genomic DNA was extracted using the phenol–chloroform–isoamyl alcohol (PCI) method. Briefly, oral swab samples were suspended in TE buffer and subjected to cell lysis using sodium dodecyl sulfate (SDS) and proteinase K to ensure efficient disruption of microbial cells and protein degradation. The resulting lysates were then extracted with phenol–chloroform–isoamyl alcohol, followed by centrifugation to separate the aqueous and organic phases. The upper aqueous phase, containing purified DNA, was carefully transferred to a new tube, and DNA was subsequently precipitated using isopropanol. The DNA pellet was washed with ethanol, air-dried, and resuspended in TE buffer for downstream applications. DNA quality and integrity were evaluated by 1% agarose gel electrophoresis, and DNA concentration was measured prior to PCR amplification. To assess bacterial DNA integrity and amplifiability prior to microbiome library preparation, the nearly full-length bacterial 16S rRNA gene (~1.5 kb) was amplified using universal primers 27F (5′-AGAGTTTGATCMTGGCTCAG-3′) and 1492R (5′-TACGGYTACCTTGTTACGACTT-3′). This PCR assay was performed solely as a quality-control step and was not used for microbiome sequencing library construction. Universal bacterial primers were selected to maximize coverage of both Gram-positive and Gram-negative taxa. PCR amplification was performed in 25 μL reaction mixtures containing template DNA, PCR master mix, forward and reverse primers, and nuclease-free water. The thermal cycling conditions consisted of an initial denaturation at 95 °C for 3 min, followed by 30 cycles of denaturation at 95 °C for 30 s, annealing at 55 °C for 30 s, and extension at 72 °C for 90 s, with a final extension step at 72 °C for 10 min. Amplified products were verified by agarose gel electrophoresis, and amplicons of the expected size (~1.5 kb) confirmed DNA integrity and were not used for microbiome sequencing library preparation. Negative controls were included during PCR amplification and showed no detectable amplification. No commercial mock community standard was included in the present study. This represents a limitation because amplification and taxonomic classification bias could not be directly assessed. PCR products were visualized using agarose gel electrophoresis (1–1.5% agarose gel). Successful amplification was confirmed by the presence of bands at approximately 1.5 kb, while negative controls showed no amplification (Figure 1A–H) [11,12].

### 2.3. Library Preparation and High-Throughput Sequencing

For microbiome sequencing, the V3–V4 hypervariable region of the bacterial 16S rRNA gene was amplified using the primer pair 341F (5′-CCTACGGGNGGCWGCAG-3′) and 805R (5′-GACTACHVGGGTATCTAATCC-3′), generating amplicons of approximately 460 bp prior to adapter addition. Purified PCR products were subsequently used for library preparation according to the sequencing platform protocol. Sequencing libraries targeting the V3–V4 region of the bacterial 16S rRNA gene were prepared according to standard amplicon sequencing protocols. Libraries were quantified, normalized, pooled, and sequenced on the Illumina NovaSeq platform using paired-end (PE) sequencing chemistry. Raw sequencing output per sample ranged from approximately 65,000 to 80,000 reads (Supplementary Table S1) [13].

### 2.4. Library Quality Assessment and Fragment Size Validation

The quality, purity, and size distribution of sequencing libraries were evaluated prior to sequencing using an Agilent 2100 Bioanalyzer (Agilent Technologies, Santa Clara, CA, USA). Electropherogram profiles were examined to confirm the presence of a dominant fragment corresponding to the expected sequence library size (~650–700 bp) and to assess potential non-specific products or primer–dimer formation (Supplementary Figure S1). Sequencing read length distributions were further assessed following data generation to verify consistency across samples. Read length profiles were visualized as frequency distributions, and the proportion of reads within the expected size range was evaluated for each sample (Supplementary Figure S2). Samples showing consistent fragment size profiles and read length distributions were retained for downstream analysis. Together, these quality control steps were performed to ensure the reliability of the sequencing dataset and to minimize potential biases associated with fragment size variability [14].

### 2.5. Sequence Processing and Quality Control

Raw sequencing data were processed using QIIME2 (version 2020.6) [15]. The workflow included quality filtering, trimming, denoising using DADA2 (version 1.26.0) [16], paired-end merging, and chimera removal. After processing, non-chimeric reads ranged from 39,634 to 65,848 per sample (Supplementary Table S1). Amplicon sequence variants (ASVs) were used for downstream analyses to achieve single-nucleotide resolution [17].

### 2.6. Taxonomic Assignment

Taxonomic assignment of ASVs was performed against the SILVA reference database (release 138) using a combined BLAST-based (version 2.9.0) and naïve Bayesian classification approach implemented in QIIME2 (version 2020.6) (classify-sklearn). The confidence threshold for taxonomic assignment was set at 0.7 according to the sequencing provider’s analysis pipeline. ASVs were generated using the DADA2 denoising algorithm. Assignments were generated at multiple taxonomic levels, including phylum and genus. Relative abundance profiles were calculated and visualized for each group. Taxonomic comparisons were performed at both phylum and genus levels to identify group-specific microbial signatures.

### 2.7. Diversity Analysis

#### 2.7.1. Alpha Diversity

Alpha diversity was assessed using the Shannon diversity index, which accounts for both species richness and evenness. Rarefaction curves were generated to evaluate sequencing depth sufficiency. Group comparisons were performed using non-parametric statistical tests due to small sample size and potential non-normality.

#### 2.7.2. Beta Diversity

Beta-diversity analyses were performed using multiple distance metrics, including Bray–Curtis, binary Jaccard, weighted UniFrac, and unweighted UniFrac distances. Bray–Curtis results are presented in the main manuscript because they provided the most interpretable visualization of community differences among study groups [18]. Differences in community composition were tested using PERMANOVA (permutational multivariate analysis of variance) [19]. Principal Coordinates Analysis (PCoA) was performed to visualize patterns of similarity and clustering among samples. Effect size (R^2^) was reported alongside *p*-values to provide a quantitative measure of group contribution to microbiome variation [20].

### 2.8. Rarefaction and Sequencing Depth Assessment

Rarefaction analysis was conducted to evaluate whether sequencing depth was sufficient to capture microbial diversity. Curves were generated by subsampling reads across increasing sequencing depths and calculating observed ASVs. The plateauing of rarefaction curves was used as evidence of adequate sampling effort.

### 2.9. Core Microbiome Analysis

Core microbiome analysis was performed to identify taxa consistently present within each group. Core ASVs were defined as those present in ≥4 out of 5 samples within a group. Overlap among groups was assessed, and shared ASVs were visualized using network analysis. Node size represented mean relative abundance, and connections indicated shared presence across groups.

### 2.10. Genus-Level Differential Abundance and Clustering Analysis

Amplicon sequence variants (ASVs) obtained from DADA2 processing were taxonomically classified using a reference database (e.g., SILVA). Relative abundance at the genus level was calculated by normalizing ASV counts to total reads per sample. For visualization, the most abundant genera across all samples were selected and plotted as mean relative abundance per group (*n* = 5 per group). Less abundant genera were included in an inset panel to allow finer comparison without distortion from dominant taxa.

### 2.11. Functional Prediction (PICRUSt2 Analysis)

Functional profiles were predicted using PICRUSt2 [21], which infers metagenomic content from 16S rRNA gene data. Predicted pathways were annotated based on KEGG (Kyoto Encyclopedia of Genes and Genomes) level 2 categories. Statistical comparisons of pathway abundances between groups were performed, and results were visualized as proportion differences with confidence intervals (Supplementary Table S8). Due to the predictive nature of PICRUSt2, results were interpreted as hypothesis-generating rather than definitive functional evidence.

### 2.12. Statistical Analysis

All analyses were performed using QIIME2 (version 2020.6.0) and R statistical software. Alpha-diversity metrics were calculated using mothur (version 1.34.4) and compared among groups using non-parametric statistical tests. Beta-diversity analyses, including Bray–Curtis, weighted UniFrac, and unweighted UniFrac distance metrics, were conducted in QIIME2, and group differences were evaluated using PERMANOVA with 999 permutations. Ordination and ecological analyses were performed using the vegan package (version 2.3-0), and graphical visualization was generated using ggplot (version 3.1.1). Differential abundance analyses at the genus level were performed using the Kruskal–Wallis test followed by false discovery rate (FDR) correction for multiple comparisons. For PICRUSt2 functional prediction analyses, KEGG Level 2 pathway comparisons were conducted in STAMP, and statistical significance was interpreted primarily on the basis of FDR-adjusted *p*-values. A significance threshold of *p* < 0.05 was applied throughout the study. Given the limited sample size (*n* = 5 per group), the results were interpreted with emphasis on effect size, biological relevance, and consistency across analytical approaches. Although animals in Groups B and C originated from the same households, the limited number of cohabiting households (*n* = 5) was insufficient to support reliable mixed-effects or household-level random-effect modeling. Therefore, all statistical analyses were performed at the individual-animal level, and the findings should be considered exploratory. In addition, both raw *p*-values and FDR-adjusted q-values were reported for genus-level comparisons to reduce the likelihood of false-positive findings associated with multiple testing.

## 3. Results

### 3.1. PCR Amplification and Sequencing Performance

The study included 20 clinically healthy companion animals (10 dogs and 10 cats) aged 2–10 years. Detailed demographic and husbandry information, including sex, age, diet, and housing conditions, is summarized in Table 1. Fragment analysis further validated standardized amplification of the target area in all samples. The electropherogram profiles exhibited a prominent peak in the desired size range of approximately 650–700 bp, with virtually no signs of nonspecific amplification or primer-dimer interaction (Figure S2). The overall distribution of DNA fragment sizes remained consistent, despite the fact that the peak intensity of some samples varied depending on the quantity of starting DNA. This indicates that the genetic libraries prepared in all experimental groups were of comparable quality. High-throughput genome sequencing yielded an extensive volume of data reads, generating around 65,000 to 80,000 raw data reads per sample. Following quality screening, noise reduction, merging, and chimera removal, the quantity of high-quality data (non-chimeric reads) per sample ranged from 39,634 to 65,848 reads (Supplementary Table S1). The results indicate that PCR amplification, library preparation, and sequencing were successfully performed and generated datasets of sufficient quality for downstream analyses.

### 3.2. Sequencing Depth Analysis

**Figure 2:**
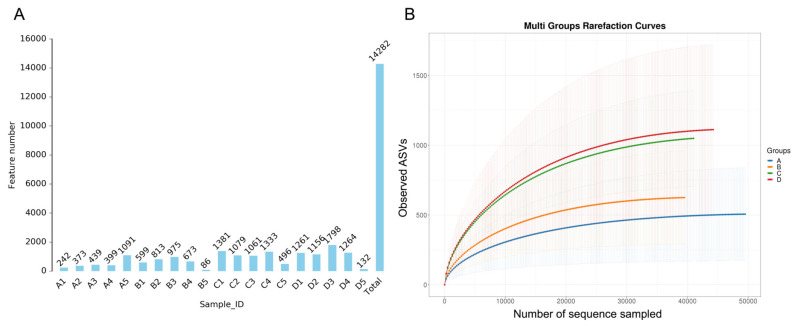
Sequencing depth and feature richness. (**A**) Number of observed amplicon sequence variants (ASVs) per sample and total ASVs across all samples. (**B**) Rarefaction curves showing the relationship between sequencing depth and observed ASVs across Groups A–D. Curves approaching saturation indicate sufficient sequencing depth to capture the majority of microbial diversity.

Analysis of sequencing data revealed substantial microbial diversity across samples. A total of 14,282 Amplicon Sequence Variants (ASVs) were identified across all samples, indicating substantial disparities in microbial richness among the experimental groups (Figure 2A). The feature counts indicated a systematic distribution of microbial diversity across the sample groups. Group D (cats housed individually) consistently exhibited the highest microbial richness, succeeded by Group C (cats cohabiting with dogs), Group B (dogs cohabiting with cats), and ultimately Group A (dogs housed individually), which displayed the lowest diversity. This trend suggests a gradient of microbial complexity that may be associated with host species and housing conditions (Figure 2B).

The rarefaction analysis was used to confirm that the genetic sequencing data reflected the true variety present. The results indicated that the rarefaction curves for all groups distinctly approached a saturation point, signifying that the depth of genetic sequencing was adequate, and more sequencing data would yield only a minimal increase in the discovery of novel microbial species (Supplementary Tables S3 and S4). The graphs for groups D and C have much higher curves than those for groups A and B, demonstrating a pronounced disparity in richness among the groups. The lack of non-saturating curves indicates the absence of under-sampling, which would not influence or introduce mistakes in following diversity comparisons (Figure 2B). These findings indicate that sequencing depth was sufficient for downstream diversity, taxonomic, and functional analyses.

### 3.3. Alpha Diversity Analysis

**Figure 3:**
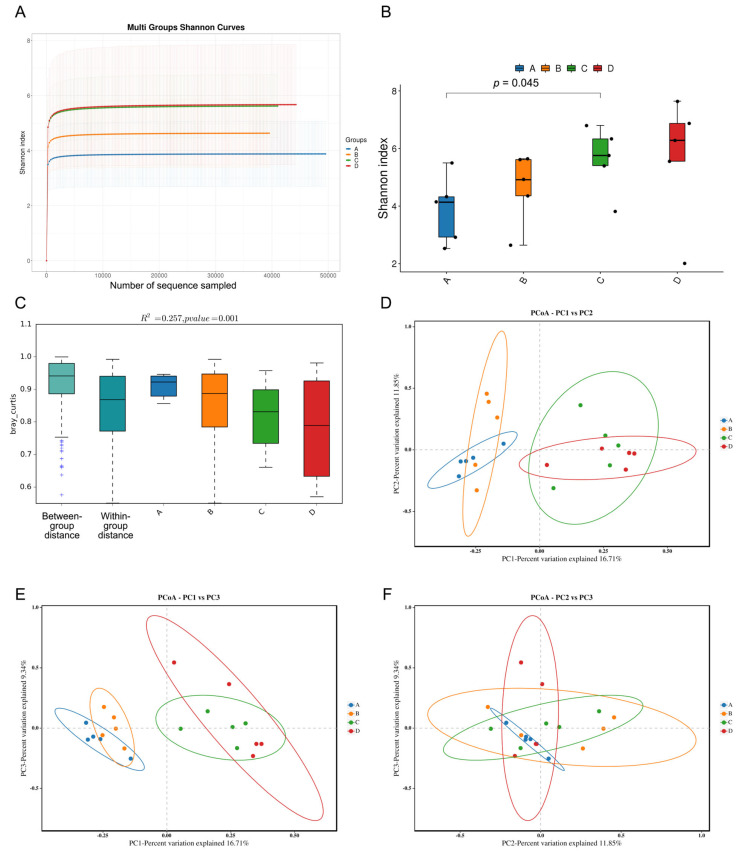
Alpha and beta diversity analyses of oral microbiome. (**A**) Rarefaction curves of the Shannon diversity index across all samples, demonstrating stabilization of diversity estimates. (**B**) Boxplot of Shannon diversity index across groups, with statistical comparison (*p* = 0.045). (**C**) Bray–Curtis dissimilarity analysis comparing within-group and between-group distances (R^2^ = 0.257, *p* = 0.001). (**D**–**F**) Principal Coordinates Analysis (PCoA) plots based on Bray–Curtis distances, showing clustering patterns across groups along different axes (PC1–PC2, PC1–PC3, PC2–PC3). Ellipses represent group-level clustering tendencies.

The Shannon diversity index was used to evaluate microbial richness and evenness within each experimental group, thereby assessing the complexity of the oral microbial community (Supplementary Table S5). The analysis indicated that the curve rapidly attained saturation and plateaued across all samples, confirming that the extent of sequencing depth is adequate for accurately assessing intra-sample diversity, and that augmenting the sequencing data would not substantially modify the diversity values (Figure 3A and Supplementary Table S6).

The Shannon Index research indicated a distinct gradient trend of rising biodiversity. Group D exhibited the most biodiversity, succeeded by groups C and B, whilst group A demonstrated the lowest biodiversity. The notable disparity in biodiversity among groups (*p* = 0.045) suggests that the settings and variables in each experimental group led to varying degrees of microbial community complexity (Figure 3B). Although statistically significant, these findings should be interpreted cautiously because the limited sample size may increase the influence of individual-level variation. Furthermore, intragroup variability was detected, particularly in group D, indicating inter-individual heterogeneity among the animals. Nonetheless, the consistent trend observed across both rarefaction curves and boxplot distributions supports the robustness of the diversity pattern. Overall, these findings indicate that microbial communities become progressively more diverse from Group A to Group D, suggesting potential ecological differences among study groups.

### 3.4. Beta Diversity and Community Structure

To assess the differences in oral microbial composition between experimental groups, the Bray–Curtis dissimilarity index was used. This revealed that animal grouping factors significantly influenced microbial composition (PERMANOVA: R^2^ = 0.257, *p* = 0.001), explaining approximately 25.7% of the variance. This represents a moderate effect size, indicating clear differentiation of microbial communities. The findings of Principal Coordinates Analysis (PCoA) elucidated the pattern, demonstrating that samples in Group A were closely clustered, indicating minimal intra-group variability, but Group B had a broader data distribution, signifying greater inter-individual variation. Groups C and D exhibit a discernible division into discrete clusters, albeit with considerable overlap, suggesting that the microbiomes of cats, whether housed singly or alongside dogs, are not entirely distinct (Figure 3D–F).

Visualization across various axes (PC1–PC2, PC1–PC3, and PC2–PC3) generally corroborated these tendencies, suggesting that group-level disparities were influenced by multivariate alterations in community composition rather than by singular dominant taxa. These discoveries were additionally corroborated by distance-based comparisons, where between-group distances were generally greater than within-group distances, although substantial overlap was observed (Figure 3C), hence reinforcing the existence of group-dependent structuring. Nevertheless, a certain extent of overlap among groups was noted, aligning with common environmental factors and intrinsic biological diversity. Supplementary investigation of UniFrac measures yielded further insights. Unweighted UniFrac (based on presence/absence) demonstrated moderate differentiation among groups, with considerable overlap in microbial composition (Supplementary Figure S3A–C).

In this research, Group B showed the greatest dispersion, but Groups C and D revealed a tendency to cluster closer, indicating commonalities in taxonomic existence. Conversely, weighted UniFrac (abundance-weighted) exhibited more distinct group differentiation (Supplementary Figure S3D–F), suggesting that fluctuations in relative abundance had a more significant role in community-level separation. Group A constituted a rather separate cluster, while Groups C and D exhibited partial overlap, indicating common dominating taxa. Group B exhibited more dispersion, indicative of heightened variability in abundance profiles. Collectively, these findings suggest that whereas microbial membership is somewhat common throughout groups, variations in relative abundance play a more significant role in community diversification. Due to the restricted sample size, these patterns ought to be regarded as descriptive and hypothesis-generating rather than conclusive.

### 3.5. Taxonomic Composition

The taxonomic structure of the oral microbiome differed among samples but was consistently dominated by a restricted number of predominant bacterial phyla (Supplementary Figure S4A). *Proteobacteria* and *Firmicutes* were the predominant phyla across all groups, although their relative abundances varied significantly among individuals. Other phyla, such as *Bacteroidota*, *Fusobacteriota*, and *Actinobacteriota*, were consistently identified at lower relative abundances in various samples, suggesting a widely shared taxonomic framework with a varied abundance structure.

**Figure 4:**
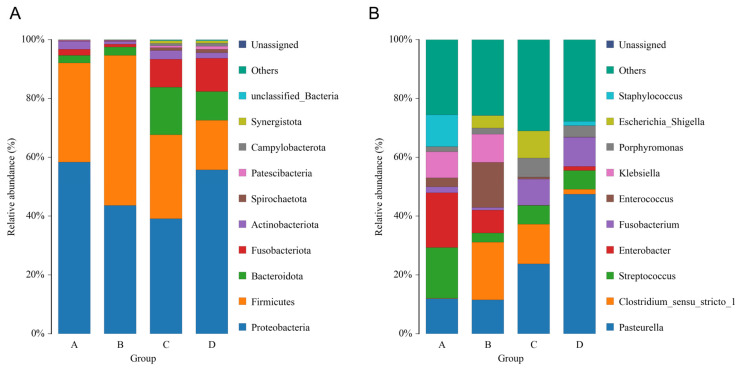
Taxonomic composition of oral microbiome. (**A**) Relative abundance of dominant bacterial phyla across groups (A–D). (**B**) Relative abundance of dominant bacterial genera across groups. Only the most abundant taxa are shown; remaining taxa are grouped as “Others.” Taxonomic profiles highlight group-specific microbial signatures.

Microbial communities were more heterogeneous by genus (Supplementary Table S7 and Figure S4B). *Pasteurella*, *Streptococcus*, *Fusobacterium*, and *Porphyromonas* were common genera, but their contributions varied greatly. A single genus (e.g., *Pasteurella* or *Clostridium_sensu_stricto_1*) dominated certain individuals, while others had more uniformly distributed communities. *Enterobacter*, *Klebsiella*, and *Escherichia/Shigella* were found in certain samples at low or fluctuating abundances. Grouping revealed phylum-level composition variations (Figure 4A). *Proteobacteria* were more abundant in Group A, while *Firmicutes* were more prevalent in Group B. Group C had a more equal distribution across different phyla, while Group D had more *Fusobacteriota* and *Bacteroidota*, taxa associated with anaerobic and polymicrobial environments. Although there was significant overlap, these patterns suggest community structure varied by housing condition. More genus-level summaries showed comparable trends (Figure 4B). Pasteurella was found in all groups, confirming its oral microbiome significance. In contrast, Groups C and D had more *Fusobacterium* and *Porphyromonas*, whereas Group B had more *Clostridium_sensu_stricto_1*. Despite these trends, no genus was uniquely connected with a group, and inter-individual variability was detected.

Although several dominant taxa belonged to Gram-negative bacterial groups, a broad range of Gram-positive taxa, including *Streptococcus*, *Staphylococcus*, *Actinomyces*, and *Clostridium*-related organisms, were also consistently detected across samples. Therefore, the observed community structure is unlikely to reflect selective recovery of Gram-negative bacteria and is more consistent with previously reported oral microbiome profiles in companion animals.

These data suggest that dogs and cats share a vast number of bacterial taxa in their oral microbiomes, with distinctions between groups driven by relative abundance rather than taxonomic presence or absence. The small sample size makes these patterns descriptive and hypothesis-generating rather than definite proof of group-specific microbial fingerprints. The following part presents further differential taxonomic statistical analysis.

### 3.6. Genus-Level Differential Abundance Analysis

Hierarchical clustering analysis indicated only limited sample clustering according to habitat circumstances, consistent with previous beta diversity analysis findings. Although clustering was not definitively evident across all samples, those within the same habitat group exhibited a tendency to cluster more tightly than those from different groups, reflecting a specific microbial structure with some existing overlap.

**Figure 5:**
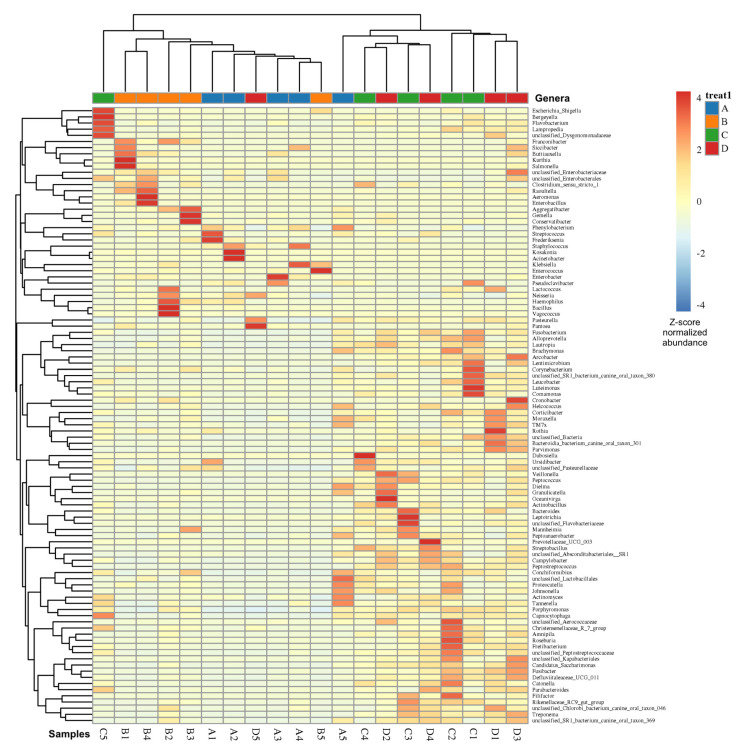
Heatmap of genus-level microbial abundance. Hierarchical clustering heatmap showing Z-score normalized relative abundance of dominant bacterial genera across all samples. Rows represent genera and columns represent samples. Clustering was performed using distance-based methods to identify patterns of similarity among samples and taxa. Color intensity reflects relative abundance (red = high, blue = low).

**Figure 6:**
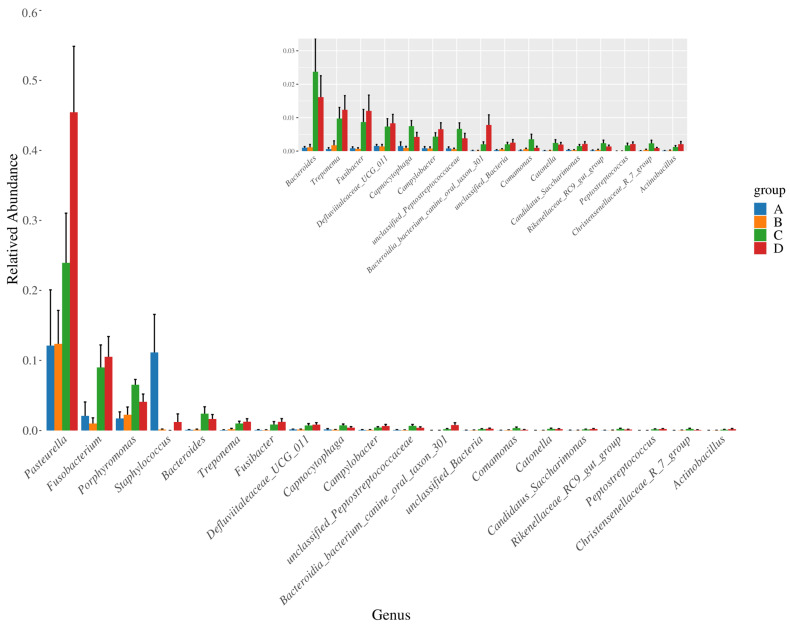
Differential genus-level relative abundance across experimental groups. Bar plots showing the relative abundance of dominant bacterial genera across Groups A–D. Each bar represents the mean relative abundance of a given genus within each group, with error bars indicating variability among biological replicates (*n* = 5 per group). The main panel highlights major genera contributing to community composition, including *Pasteurella*, *Fusobacterium*, *Porphyromonas*, and *Staphylococcus*. The inset panel provides a higher-resolution comparison of less abundant genera across groups. Group-specific patterns are evident, with Group D showing marked enrichment of *Fusobacterium*, while Group C exhibits intermediate abundance, and Groups A and B show comparatively lower levels. Differences in other genera, including *Treponema*, *Campylobacter*, and *Peptostreptococcus*, further illustrate compositional shifts across groups.

A comparison of relative abundance proportions revealed interesting findings among several bacterial genera in Figure 5 and Figure 6. *Pasteurella* presented a relatively consistent proportion across all experimental groups (approximately 10–13%), confirming its role as a fundamental and important component of the oral microbiome in animals. *Fusobacterium* exhibited a distinct gradient increase from group A to group D, peaking at 45% in group D, in contrast to 2–4% in groups A and B, and a moderate 23% in group C; this trend represents one of the most significant alterations in the dataset. This may indicate a transition in the microbial community composition towards anaerobic or biofilm-associated species. *Porphyromonas* exhibited greater prevalence in groups C and D, while *Staphylococcus* was predominant in group B (dogs cohabiting with cats), indicating a proliferation of facultative bacteria that varies according to environmental factors.

**Figure 7:**
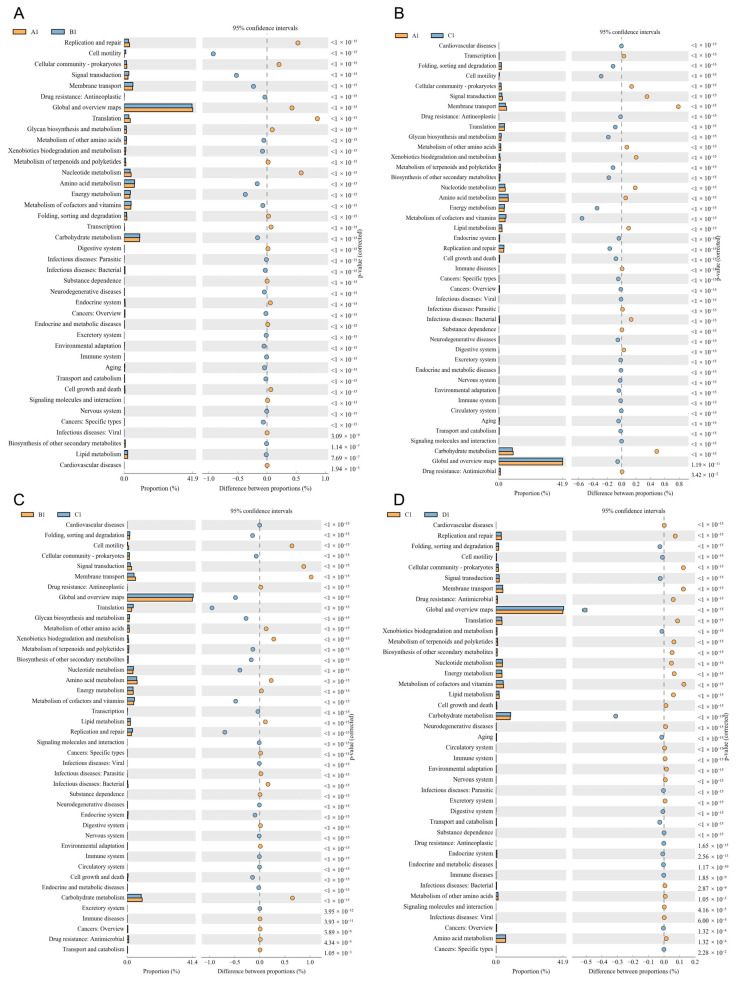
Functional prediction of microbial communities (PICRUSt2 analysis). (**A**–**D**) Differential abundance of KEGG level 2 pathways between selected group comparisons (A vs. B, A vs. C, B vs. C, C vs. D). Bar plots represent pathway proportions, and dot plots indicate differences between groups with 95% confidence intervals. *p*-values are shown for each pathway comparison. Functional categories include metabolism, cellular processes, environmental information processing, and disease-related pathways.

In addition to the dominant group, lower-density bacteria, including *Treponema*, *Campylobacter*, and *Peptostreptococcus*, are frequently identified or exhibit increased densities in groups C and D. The proliferation of particular bacterial groups enhances the complexity of the microbial community in felines relative to other species. Genus-level analysis reveals that variations in microbial structure are influenced not only by overall diversity but also by substantial alterations in the density of particular bacterial groups. Differential abundance analysis identified several genera showing apparent differences in relative abundance among groups (Figure 7 and Supplementary Table S7). Genera such as *Porphyromonas*, *Enterobacter*, and several low-abundance taxa contributed to the observed compositional variation between canine and feline oral microbiomes. However, after correction for multiple testing using the false discovery rate (FDR) method, no taxonomically classified genus remained statistically significant (FDR-adjusted *p* < 0.05). Therefore, genus-level differences were interpreted descriptively and considered hypothesis-generating observations rather than definitive taxonomic associations.

### 3.7. Functional Prediction of Microbial Metabolic Potential

Functional genome profiling research utilizing PICRUSt2 was applied to comprehend the functional implications linked to taxonomic variance (Supplementary Table S8 and Figure 7A–D). The findings indicated that all experimental groups displayed uniform core functional categories, encompassing membrane transport, signal transduction, replication and repair, as well as glucose and amino acid metabolism, which are vital processes for the survival of oral bacteria.

Despite having similar core functional structures, significant differences in the proportion of metabolic pathways were found between groups, particularly in groups C and D, which showed an increasing trend in pathways related to metabolic processes and biosynthetic functions, suggesting higher functional complexity compared to groups A and B. Furthermore, Group D showed a higher proportion of pathways involved in DNA replication and repair, as well as energy metabolism, compared to other groups. This may indicate specific microbial growth dynamics or adaptive mechanisms within this group.

Pairwise comparisons identified several KEGG pathways that remained significantly different after FDR correction (FDR-adjusted *p* < 0.05), consistent with previously identified taxonomic differences. Interestingly, variations were found in pathways related to membrane transport and other metabolic functions, which may be linked to differences in antimicrobial tolerance or stress response across different habitats (Supplementary Table S8 and Figure 7A–D). Because these predictions were inferred from 16S rRNA gene data, they should be interpreted as hypothesis-generating rather than direct evidence of functional activity.

### 3.8. Core Microbiome Analysis and Interspecies Microbial Sharing Patterns

**Figure 8:**
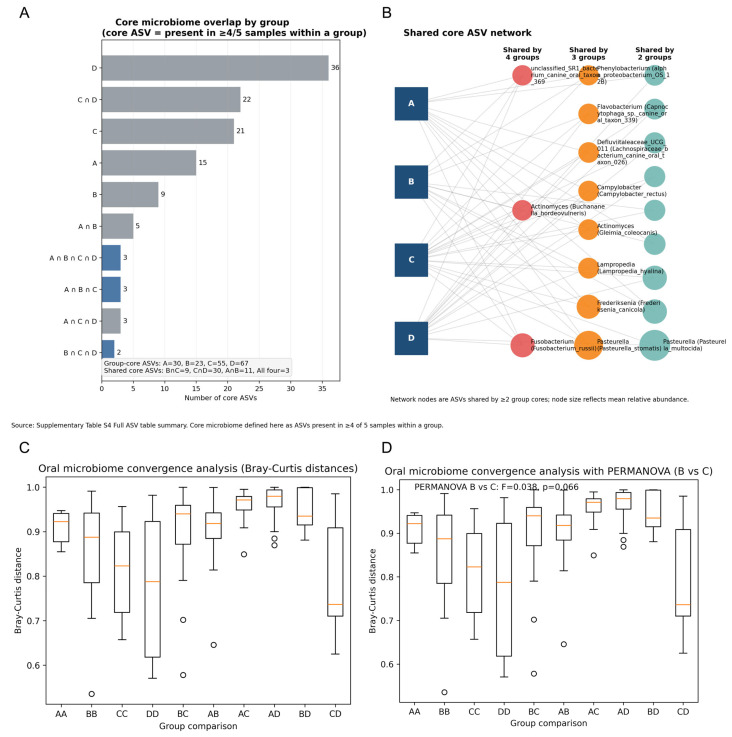
Core microbiome and shared taxa network. (**A**) Core microbiome overlap across groups, defined as ASVs present in ≥4 out of 5 samples within a group. Bars represent the number of core ASVs unique to or shared between groups. (**B**) Network visualization of shared core ASVs across groups. Nodes represent ASVs, and edges indicate presence across multiple groups. Node size reflects mean relative abundance. (**C**) Bray–Curtis distance comparisons among group combinations, illustrating microbiome convergence patterns. (**D**) PERMANOVA analysis comparing selected groups (e.g., B vs. C), showing group-level differences in community composition.

This study conducted a core microbiome analysis to identify consistently present microbial features (ASVs) within each group, based on the criterion of detecting amplicon sequence variants (ASVs) in a minimum of 4 out of 5 samples per group (≥80%). The study results indicated significant differences in the number of core ASVs among the experimental groups (Figure 8A). Group D exhibited the largest core microbiome (36 ASVs), followed by Group C (21 ASVs) and Group A (15 ASVs), whereas Group B showed the smallest core (9 ASVs). The observed pattern indicates that groups C and D have more stable microbial communities characterized by consistent intragroup sharing, whereas group B demonstrated greater inter-individual variability. Overlap analysis and microbial network structure analysis showed that only a few ASVs were shared by more than one group. In fact, only 3 ASVs were found in all 4 experimental groups. This might indicate a limited universal core microbiome across groups (Figure 8A). Most core ASVs were group-specific or shared among only two to three groups, highlighting the influence of group-specific ecological factors on microbiome composition.

Network-based visualization indicates that microorganisms shared across multiple groups formed interconnected clusters, which include dominant genera commonly found in animal mouths, such as *Pasteurella*, *Actinomyces*, and *Fusobacterium*. This finding indicated the presence of a conserved microbial backbone across hosts. Network analysis further showed that these shared ASVs formed connections between groups B and C, suggesting the presence of common microbial members. Representative shared ASVs included oral-associated taxa such as *Porphyromonas canoris*, *Porphyromonas gulae*, and *Alloprevotella*, together with taxa commonly associated with enteric or opportunistic bacteria, including *Clostridium perfringens*, *Leclercia adecarboxylata*, and *Klebsiella pneumoniae* (Figure 8B and Supplementary Table S9). Despite being shared, several ASVs displayed differences in relative abundance between groups B and C. For example, *Leclercia adecarboxylata* (ASV3042; log10 fold difference = +1.42), *Klebsiella pneumoniae* (ASV3699; +1.48), and *Enterobacteriaceae*-related taxa (ASV3051; +2.18) were more abundant in group B, whereas oral-associated taxa such as *Porphyromonas canoris* (ASV3736; −1.68) and *Alloprevotella* sp. (ASV3765; −1.45) were more abundant in group C. Some taxa, including *Clostridium perfringens* (ASV4764; +0.16), showed relatively similar abundance between groups. These observations indicate that microbiome overlap does not necessarily correspond to identical community structure. Conversely, less group-specific taxonomies may reflect differences specific to the host, environment, or interactions within the microbiome composition. To evaluate the impact of cohabitation on microbiome similarity, Bray–Curtis distances showed substantial overlap between intra- and inter-group comparisons, indicating heterogeneous patterns of microbial similarity rather than strictly group-specific clustering (Figure 8C). Comparisons among certain groups (e.g., A–C and A–D) showed separation patterns, although certain pairs exhibited partial overlap, suggesting varied patterns of microbial community convergence. PERMANOVA analysis (B vs. C) indicated no statistically significant difference in community composition (F = 0.038, *p* = 0.066), despite partial overlap in Bray–Curtis distances (Figure 8D). This observation may suggest a tendency toward microbiome convergence under shared household conditions. However, because dogs and cats possess distinct host-associated physiological characteristics that strongly influence oral microbial community structure, these findings should not be interpreted as definitive evidence of interspecies microbial sharing.

## 4. Discussion

This study provides one of the first characterizations of the oral microbiome of cohabiting dogs and cats in Thailand and describes differences in microbial diversity, community composition, core microbiome structure, and predicted functional profiles among companion animals living under different housing conditions [22,23,24,25]. A notable strength of the study is that animals in the cohabiting groups (Groups B and C) were sampled from the same multi-pet households, thereby reducing household-level environmental variability and allowing a more direct comparison between dogs and cats exposed to similar living conditions. The observed microbiome patterns may therefore reflect a combination of host-associated biological characteristics and shared environmental influences. However, because factors such as diet, age, breed, and household management practices were not evaluated as independent variables, their relative contributions cannot be determined from the present study.

A progressive increase in microbial diversity was observed from Group A to Group D. Although greater diversity is often considered a characteristic of stable microbial ecosystems, diversity alone does not necessarily indicate a healthier microbiome [26,27]. In the present study, Groups C and D exhibited higher relative abundances of *Fusobacterium* and *Porphyromonas*, genera commonly associated with polymicrobial biofilms, anaerobic environments, and periodontal disease [28]. These findings may suggest a shift toward a more complex biofilm-associated community structure. In contrast, genera such as *Pasteurella* and *Actinomyces* were consistently detected across all groups, indicating the presence of a conserved oral microbial framework among companion animals. Nevertheless, because oral health parameters were not assessed systematically, these observations should be interpreted cautiously and considered descriptive rather than diagnostic [29,30,31].

Core microbiome analysis revealed limited overlap among groups, with only a small number of ASVs shared across all animals. Most core ASVs were group-specific or shared among a limited subset of groups, highlighting the individualized nature of oral microbial communities. Interestingly, several oral-associated taxa, including *Porphyromonas* spp. and *Alloprevotella* spp., were shared between cohabiting dogs and cats, suggesting the presence of shared microbial members between cohabiting animals. Similar patterns of microbiome sharing have been reported previously in humans and companion animals living in close contact [32,33,34]. Although these findings do not constitute direct evidence of microbial transmission, they support the concept that cohabitation may contribute to microbiome convergence and underscore the relevance of companion animal microbiome research within a One Health framework.

Functional prediction using PICRUSt2 suggested differences in pathways related to membrane transport, replication and repair, and general metabolic functions. These pathways are commonly associated with microbial adaptation, ecological competition, and biofilm development [35,36]. However, because PICRUSt2 provides only inferred functional profiles based on 16S rRNA gene data, these findings should be regarded as hypothesis-generating rather than direct evidence of functional activity. Future shotgun metagenomic or metatranscriptomic studies will be necessary to validate these predicted functional differences and to further investigate their potential relevance to antimicrobial resistance and host–microbe interactions [37].

Several limitations should be considered when interpreting the present findings. First, the sample size was intentionally limited (*n* = 5 per group) because of strict inclusion criteria and the exploratory nature of this pilot study, reducing statistical power and limiting the ability to capture the full extent of oral microbiome variability among companion animals. Second, several potentially relevant host-related variables, including breed, neuter status, body condition score, probiotic supplementation, and detailed dental health parameters, were not systematically evaluated. Third, animals in Groups B and C originated from the same households, introducing potential household-level dependence while simultaneously reducing environmental heterogeneity. Because only five households were included, mixed-effects modeling could not be applied to separate household effects from host-species effects. Fourth, a commercial mock community was not included, preventing direct assessment of amplification and taxonomic assignment bias. Homogeneity of multivariate dispersion was not evaluated and therefore PERMANOVA results should be interpreted cautiously. Finally, no genus remained statistically significant after FDR correction, highlighting the exploratory and hypothesis-generating nature of the genus-level findings. Consequently, the present study should be regarded as a pilot investigation providing baseline data for future larger-scale and longitudinal studies. In addition, animals in Groups A and D originated from single-species households, and therefore household-specific environmental factors may have contributed to some of the observed microbiome patterns. Consequently, species effects and household effects could not be fully disentangled in the present study.

## 5. Conclusions

This exploratory study suggests that the oral microbiome of companion animals is a dynamic and context-dependent ecosystem shaped by both host-associated and environmental factors. Despite the limited sample size, differences in microbial diversity, community composition, core microbiome structure, and predicted functional profiles were observed among dogs and cats living under different housing conditions. Collectively, these findings suggest that host species remains a primary determinant of oral microbiome structure, while cohabitation may contribute to limited microbial convergence among companion animals sharing the same household environment. Although the present results should be interpreted as hypothesis-generating, they provide baseline data for future investigations of companion animal oral microbiomes within a One Health framework. Larger longitudinal and multi-household studies will be required to confirm these observations and further clarify the relative contributions of host and environmental factors.

## Figures and Tables

**Table 1:** Individual characteristics of enrolled animals.

Sample ID	Household ID	Species	Housing Condition	Sex	Age (Years)	Diet	Health Status	Recent Antibiotic Exposure
A1	H1	Dog	Single-pet household	Male	6	Commercial dry dog food	Healthy	No
A2	H1	Dog	Single-pet household	Male	6	Commercial dry dog food	Healthy	No
A3	H1	Dog	Single-pet household	Female	2	Commercial dry dog food	Healthy	No
A4	H1	Dog	Single-pet household	Female	2	Commercial dry dog food	Healthy	No
A5	H1	Dog	Single-pet household	Male	2	Commercial dry dog food	Healthy	No
B1	H2	Dog	Multi-pet household (cohabiting with cat)	Male	3	Commercial dry dog food and occasional treats	Healthy	No
B2	H3	Dog	Multi-pet household (cohabiting with cat)	Female	10	Commercial dry dog food and occasional treats	Healthy	No
B3	H4	Dog	Multi-pet household (cohabiting with cat)	Female	4	Commercial dry dog food and occasional treats	Healthy	No
B4	H5	Dog	Multi-pet household (cohabiting with cat)	Female	2	Commercial dry dog food and occasional treats	Healthy	No
B5	H6	Dog	Multi-pet household (cohabiting with cat)	Male	5	Commercial dry dog food and occasional treats	Healthy	No
C1	H2	Cat	Multi-pet household (cohabiting with dog)	Male	3	Commercial dry cat food and occasional treats	Healthy	No
C2	H3	Cat	Multi-pet household (cohabiting with dog)	Female	10	Commercial dry cat food and occasional treats	Healthy	No
C3	H4	Cat	Multi-pet household (cohabiting with dog)	Female	6	Commercial dry cat food and occasional treats	Healthy	No
C4	H5	Cat	Multi-pet household (cohabiting with dog)	Male	4	Commercial dry cat food and occasional treats	Healthy	No
C5	H6	Cat	Multi-pet household (cohabiting with dog)	Male	5	Commercial dry cat food and occasional treats	Healthy	No
D1	H7	Cat	Single-pet household	Male	10	Commercial dry cat food and occasional treats	Healthy	No
D2	H7	Cat	Single-pet household	Male	10	Commercial dry cat food and occasional treats	Healthy	No
D3	H8	Cat	Single-pet household	Female	9	Commercial dry cat food and occasional treats	Healthy	No
D4	H9	Cat	Single-pet household	Female	6	Commercial dry cat food	Healthy	No
D5	H10	Cat	Single-pet household	Female	3	Commercial dry cat food	Healthy	No

Notes: All animals were indoor companion animals maintained under stable household conditions and received species-appropriate commercial diets. Animals were clinically healthy at the time of sampling, showed no apparent oral or systemic disease, and had no recent history of antibiotic administration. Groups B and C were derived from the same five multi-pet households, with one dog and one cat sampled from each household. To minimize short-term dietary effects on the oral microbiome, samples were collected before the animals’ morning meal using a standardized sampling protocol. Household ID indicates the household of origin. Animals within the same household shared the same housing environment. Group A animals originated from the same dog-only household, Group D animals originated from a cat-only household, whereas Groups B and C were derived from five cohabiting dog–cat households.

## Data Availability

All data generated or analyzed during this study are included in the article and are available from the corresponding author upon reasonable request.

## Supplementary Materials

The following supporting information can be downloaded at https://www.mdpi.com/article/10.3390/ani16121882/s1. Table S1: Sequencing data processing summary. This table summarizes the number of sequencing reads retained at each stage of the bioinformatics workflow for all samples. The steps include raw reads, reads remaining after quality filtering (clean reads), denoised reads after error correction and ASV inference, merged paired-end reads, and the final set of non-chimeric reads. Overall, a high proportion of reads was retained throughout the pipeline, indicating good sequencing quality and minimal data loss. The final number of non-chimeric reads ranged from 39,634 to 65,848 per sample, providing sufficient depth for downstream microbiome analyses; Table S2: Read length distribution across all samples. This table presents the distribution of sequencing read lengths for each sample. Read counts are grouped by length intervals to evaluate fragment size consistency after sequencing and processing. Most reads fall within the expected amplicon size range (~650–700 bp), suggesting successful amplification and consistent sequencing performance across samples; Table S3: Rarefaction curve data. This table contains the data used to generate rarefaction curves, showing the number of observed ASVs as sequencing depth increases. These data allow assessment of whether sequencing depth was sufficient to capture the majority of microbial diversity within each sample; Table S4: Full ASV table summary. This table provides the complete ASV dataset, including taxonomic assignments and abundance counts for each sample. It serves as the foundation for all downstream analyses, including diversity estimation, taxonomic profiling, core microbiome analysis, and differential abundance testing; Table S5: Alpha diversity metrics. This table summarizes alpha diversity values calculated for each sample, including metrics such as Shannon diversity and richness. These indices were used to compare within-sample diversity across groups; Table S6: Shannon diversity curve data. This table contains the data used to generate Shannon diversity curves across sequencing depths. These data support the stability of diversity estimates and help confirm that sequencing depth was sufficient for reliable diversity analysis; Table S7: Differential genus abundance analysis (ANOVA). This table presents the results of genus-level comparisons across groups using ANOVA. It includes relative abundance values along with corresponding statistical outputs (e.g., *p*-values and, where applicable, adjusted *p*-values), providing detailed support for differences observed at the genus level; Table S8: PICRUSt2 KEGG Level 2 functional comparison. This table summarizes the statistical comparison of predicted functional pathways (KEGG Level 2 categories) generated using PICRUSt2. It includes pathway proportions, differences between groups, and associated significance values, supporting the interpretation of group-related variation in predicted microbial functional potential; Table S9: Key shared ASVs between groups B and C ranked by abundance and differential enrichment. Only ASVs detected in both groups B and C and absent in groups A and D are shown. Total abundance represents the sum of read counts across groups B and C. Log10 fold difference was calculated as log10((B + 1)/(C + 1)), where positive values indicate higher abundance in group B and negative values indicate higher abundance in group C. Selected ASVs represent dominant and biologically relevant taxa contributing to the observed microbiome overlap; Figure S1: DNA fragment analysis and library quality assessment. Representative electropherogram profiles of amplified 16S rRNA gene libraries from individual samples across all groups (A–T). Each panel shows fragment size distribution generated by capillary electrophoresis, including lower marker (LM), upper marker (UM), and the target amplicon peak (~650–700 bp). The consistent presence of a dominant peak within the expected size range indicates successful amplification and library preparation across samples; Figure S2: Length distribution of sequencing reads. Distribution of sequencing read lengths across all samples (A–T). Histograms show the frequency of reads within defined length intervals (bp). Most reads were concentrated within the expected amplicon size range, with limited variability among samples; Figure S3: Beta diversity analysis of oral microbiome using UniFrac distance metrics. (A–C) Unweighted UniFrac (presence/absence-based) and (D–F) weighted UniFrac (abundance-weighted) principal coordinate analysis (PCoA) plots comparing microbial community structure across groups: dogs housed alone (Group A), dogs cohabiting with cats (Group B), cats cohabiting with dogs (Group C), and cats housed alone (Group D). Panels show pairwise combinations of principal coordinates (PC1 vs. PC2, PC1 vs. PC3, and PC2 vs. PC3). Ellipses represent the dispersion of samples within each group. The percentage of variation explained by each axis is indicated; Figure S4: Taxonomic composition of the oral microbiome across individual samples. (A) Relative abundance of bacterial taxa at the phylum level across all samples (A1–D5). (B) Relative abundance of dominant genera across all samples, with low-abundance taxa grouped as “Others”. Each bar represents an individual sample, grouped according to housing condition: dogs housed alone (Group A), dogs cohabiting with cats (Group B), cats cohabiting with dogs (Group C), and cats housed alone (Group D). Taxonomic assignment was performed using a reference database, and relative abundances were calculated based on normalized sequence counts. Only major taxa are shown, while less abundant taxa are aggregated into the “Others” category for clarity.
