# Supplementary material for: Characterization of the Oral Microbiome and Anticipated Functional Profiles of Companion Animals in Private and Cohabiting Environments: A Pilot Study
Source: Animals (Basel). 2026 Jun 17;16(12):1882. doi: 10.3390/ani16121882 (PMC13295344; doi:10.3390/ani16121882)
Supplement: Supplementary file 1 [file animals-16-01882-s001.zip › animals-4304850-supplementary/Supplementary Final/Supplementary Table S1 Summary on raw data processing.pdf]

Supplementary Table S1 Summary on raw data processing

sample	Raw Reads	Clean Reads	Denoised Reads	Merged Reads	Non-chimeric Reads
A1	79,942	72,307	72,236	70,846	59,250
A2	79,746	73,090	72,841	70,384	49,866
A3	80,105	73,202	73,115	71,490	59,846
A4	79,995	73,099	72,941	70,956	58,399
A5	79,998	73,070	72,739	68,323	55,640
B1	65,478	59,635	59,494	57,473	39,634
B2	79,945	72,897	72,667	69,574	45,440
B3	79,220	71,917	71,615	67,508	46,937
B4	70,476	64,124	63,792	61,497	45,036
B5	79,757	73,297	73,283	72,706	60,895
C1	79,784	72,980	72,673	67,475	55,033
C2	64,289	58,390	58,048	53,622	41,360
C3	79,865	73,259	72,925	68,193	53,226
C4	80,021	73,171	72,827	68,833	55,683
C5	80,035	72,941	72,784	69,729	58,748
D1	79,988	73,574	73,247	67,526	56,743
D2	65,223	59,234	58,913	54,932	44,614
D3	78,773	72,188	71,583	65,884	46,163
D4	80,016	72,873	72,533	67,393	56,917
D5	80,003	73,201	73,175	72,626	65,848
