# Supplementary figures and images for: Characterization of the Oral Microbiome and Anticipated Functional Profiles of Companion Animals in Private and Cohabiting Environments: A Pilot Study
Source: Animals (Basel). 2026 Jun 17;16(12):1882. doi: 10.3390/ani16121882 (PMC13295344; doi:10.3390/ani16121882)

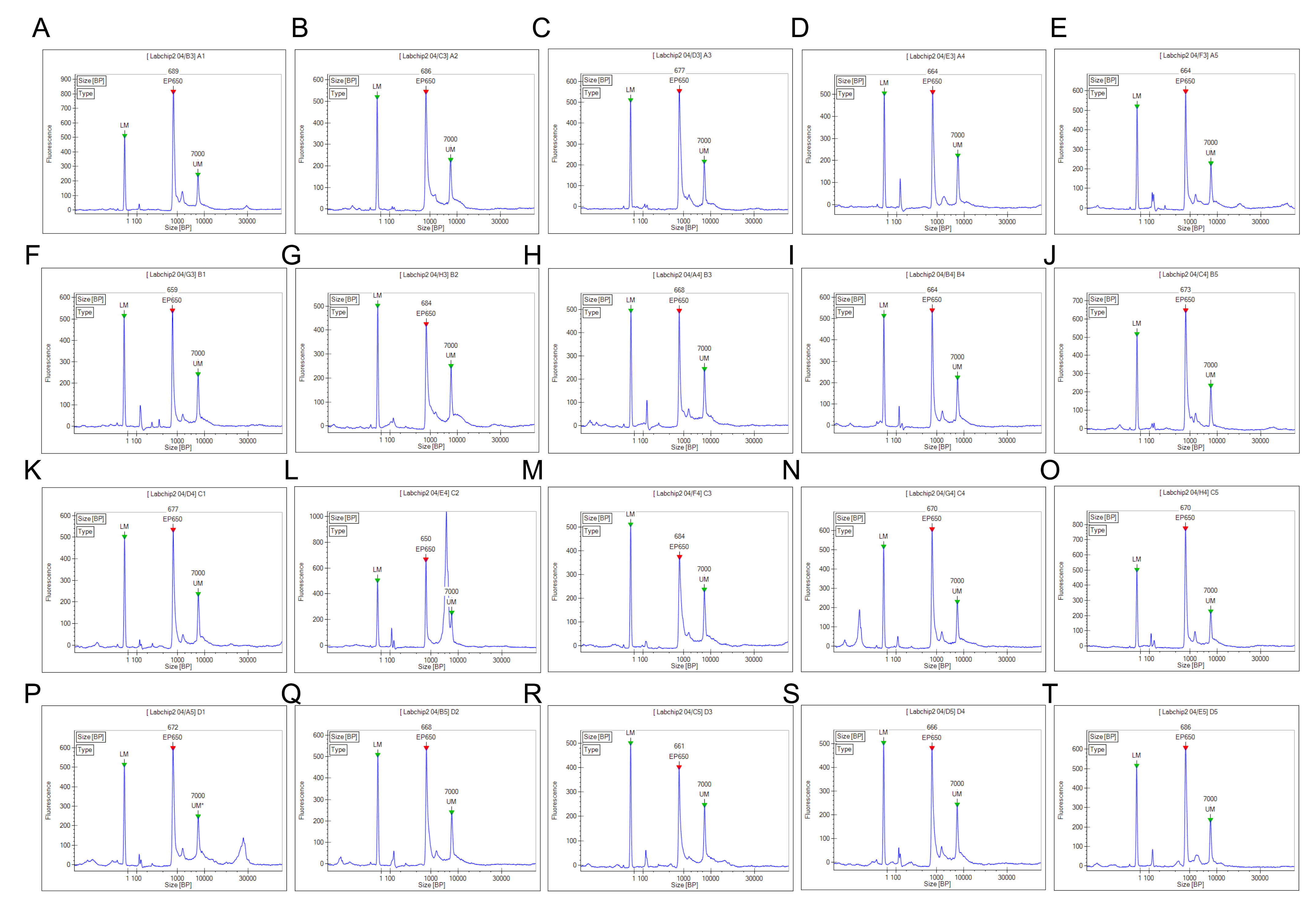

Supplement: Supplementary file 1 [file animals-16-01882-s001.zip › animals-4304850-supplementary/Supplementary Final/Figure S1.tiff]

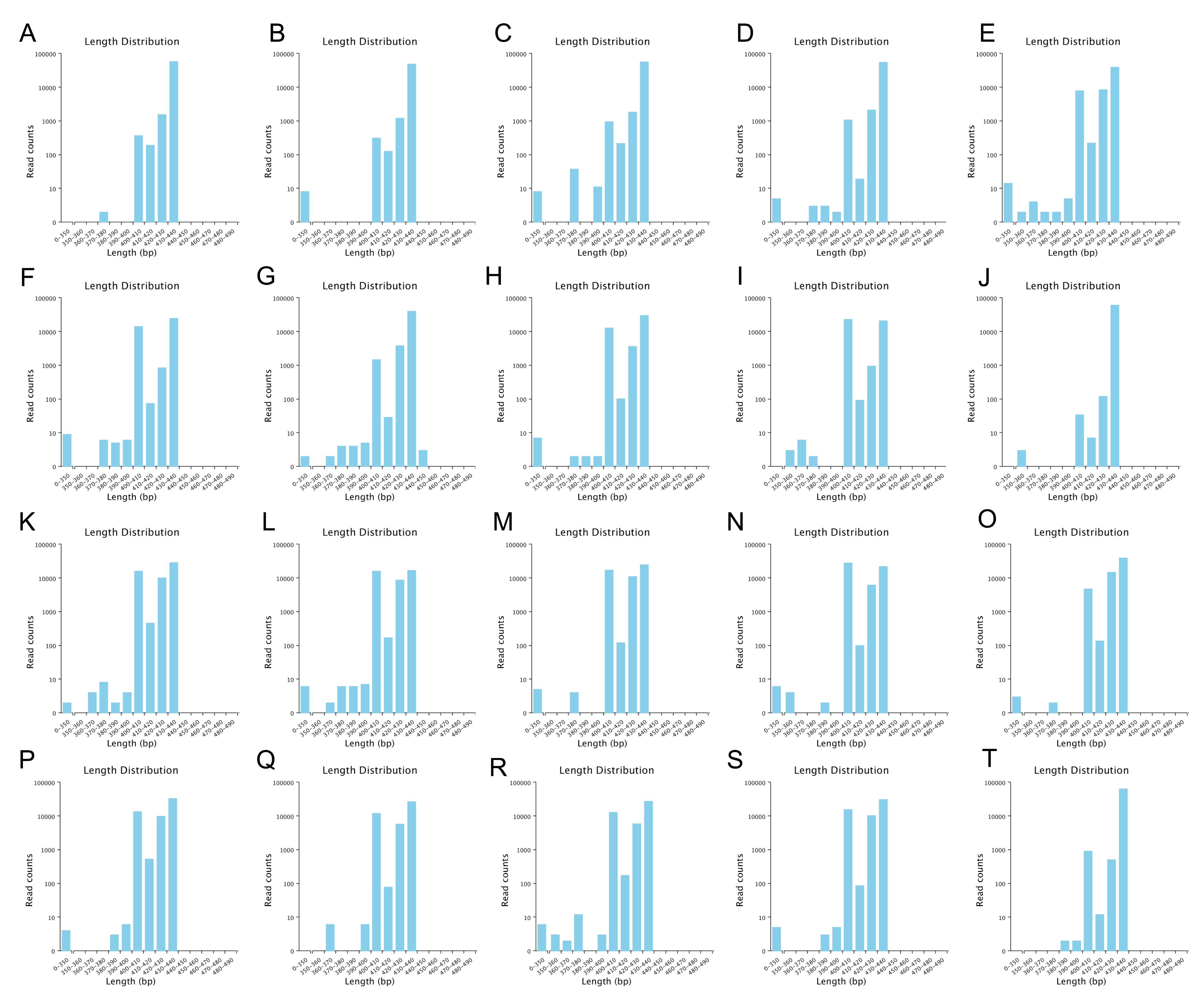

Supplement: Supplementary file 1 [file animals-16-01882-s001.zip › animals-4304850-supplementary/Supplementary Final/Figure S2.tiff]

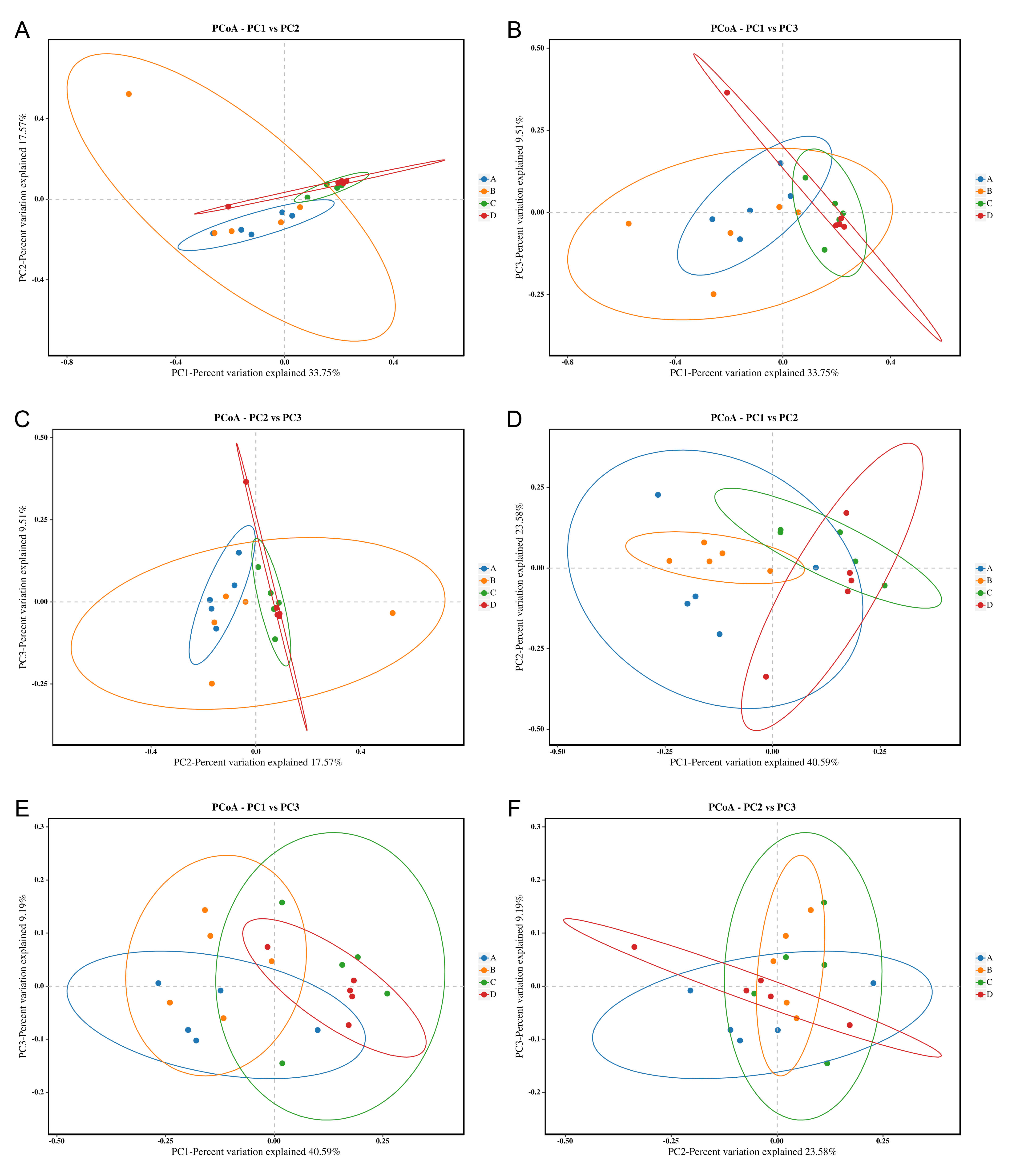

Supplement: Supplementary file 1 [file animals-16-01882-s001.zip › animals-4304850-supplementary/Supplementary Final/Figure S3.tiff]

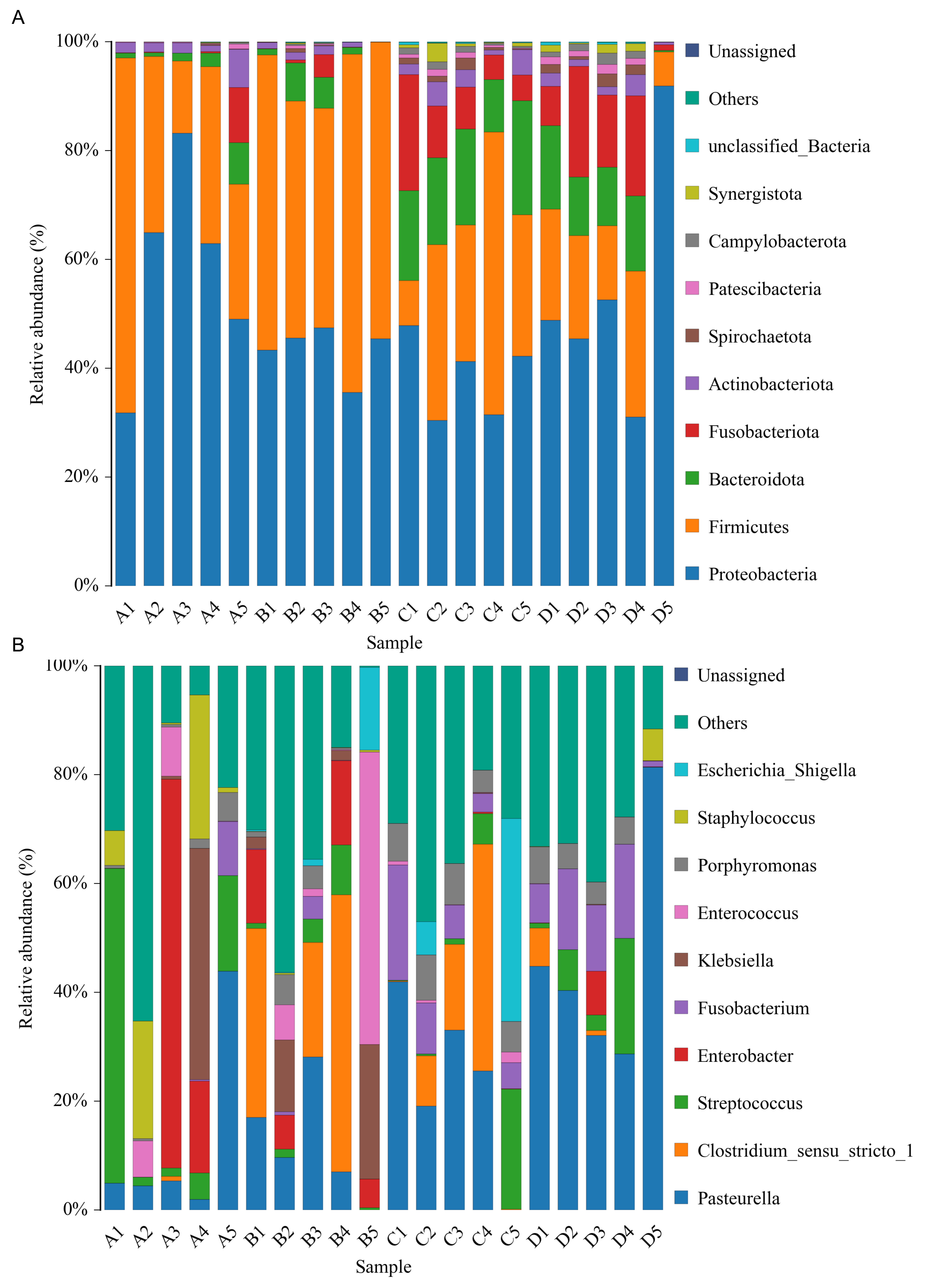

Supplement: Supplementary file 1 [file animals-16-01882-s001.zip › animals-4304850-supplementary/Supplementary Final/Figure S4.tiff]
